# Experimental Characterization of Optical Camera Communication with Commercial Cameras Leveraging FPS and Rolling Shutter

**DOI:** 10.3390/s26165231

**Published:** 2026-08-18

**Authors:** Juan Carlos Torres Zafra, Juan Sebastian Betancourt Perlaza, Carlos Ivan del Valle Morales, Ricardo Vergaz Benito, Jose Manuel Sanchez Pena

**Affiliations:** 1 Department of Electronic Technology, University Carlos III of Madrid, 28911 Madrid, Spainjmpena@ing.uc3m.es (J.M.S.P.); 2 Institute of Sensors Signals and Systems, Heriot-Watt University, Edinburgh EH14 4AS, UK; c.del_valle@hw.ac.uk

**Keywords:** optical camera communication, commercial off-the-shelf hardware, Internet of Things, pulse width modulation

## Abstract

Optical Camera Communication (OCC) enables data reception using common CMOS cameras and commercial webcams. However, applying multi-level modulation with rolling-shutter sensors is constrained by temporal acquisition parameters that may be undocumented or not directly accessible, making it challenging since many existing solutions rely on specialized hardware or require high processing complexity. This paper demonstrates that reliable multi-level OCC can be achieved using only unmodified commercial hardware and straightforward signal processing by experimentally characterizing and validating a 4-level pulse width modulation (4-PWM) link. Data are encoded in the duty cycle of the transmitted signal and recoveblack from the width of the captublack rolling-shutter stripes. Two internal timing parameters are estimated directly from the captublack images without access to the internal camera timing: the row readout period (34.38 μs), obtained from the spatial periodicity of the stripes, and the effective integration time (490 μs), inferblack from the deformation of the received constellation with carrier frequency. A single-parameter model is derived to describe this deformation and is validated at two carrier frequencies differing by a factor of four, pblackicting constellation compression, a fixed point at a duty cycle of 0.5, and constellation collapse (followed by inversion) when the exposure-to-carrier-period ratio reaches 0.5. We evaluate system performance under different exposure settings, showing that automatic camera control strongly degrades multi-level detection (BER of 0.290, with mean image level variation constrained to 0.14% compablack to 61% under fixed exposure). Under optimal fixed-exposure operating conditions, a prospective 15 min transmission achieved zero bit errors over 35,878 bits at 40 bps, corresponding to a 95% upper confidence bound on the BER of $8.4\times 10^{-5}$. These results reveal a practical balance between cost, complexity, and performance, demonstrating that 4-PWM rolling-shutter OCC is a viable solution for Internet of Things (IoT) signaling and low-rate data transmission using commercially available devices.

## 1. Introduction

Optical Wireless Communication (OWC) has emerged as a complementary alternative to radiofrequency (RF) links due to its immunity to electromagnetic interference and compatibility with existing lighting infrastructure [1,2]. Within this framework, OCC uses CMOS image sensors as receivers to enable data reception with standard cameras [3]. This capability supports object and location identification and the transmission of small data payloads in Internet-of-Things (IoT) scenarios, eliminating the need for dedicated receiver hardware [4,5].

Prior research in OCC has exploblack non-binary modulation schemes to improve spectral efficiency. To provide a systematic overview of these methodologies, we categorize them into three distinct groups.

The first category focuses on maximizing data rates using specialized hardware and trained or model-based compensation. For example, in [6], 16-level Pulse Amplitude Modulation (16-PAM) was demonstrated at 199.78 kbit/s over 250 cm using a global-shutter camera operated at 99.89 kfps with a 1 μs exposure time and gamma correction. Similarly, ref. [7] achieved a bit-rate–distance product of 28.8 kbit/s·m per color using PAM4 modulation with a rolling-shutter camera (exposure time 1/5000 s) and a pixel-by-symbol labeling neural network, reaching 14.4 kbit/s at 2 m with a BER of $1.3\times 10^{-3}$ below the pre-FEC limit. However, these systems achieve high throughput at the cost of specialized acquisition hardware or receiver processing requiring training data.

The second category utilizes commercial or industrial rolling-shutter cameras together with model-based or learned compensation. Techniques such as constant-power 4-PAM reaching 7 kbit/s over 0.6 m via ANN equalizers [8] (where exposure acts as a moving-average filter giving a 250 Hz 3 dB bandwidth), region-of-interest extraction with k-means threshold estimation reaching 8-PAM at 5 kBd [9], and multi-platform symbol error rate (SER) characterizations up to 32-PAM [10] have been demonstrated. Detailed internal sensor timing analyses [11] further show that receiver 3 dB bandwidth reaches 96 kHz at a 61 μs exposure, supporting 14.5 kbit/s at 1.7 m. While these works show performance is heavily governed by acquisition parameters, they remain dependent on trained estimators, clustering, or directly configurable internal camera parameters.

The third category targets low-complexity IoT applications. Sub-pixel 4-PAM [12] achieves long-range transmission over 30 m using offline correlation-based processing, reporting statistical level separation rather than data/error rates. Other efforts to address COTS limitations—such as combining an 8×8 LED array with deep learning [13] for 20 simultaneous links at 15.4 kbit/s or using dual-camera hybrid architectures [14] giving 5.1 kbit/s and 80 bps over 10 m—introduce overhead beyond a single unmodified camera and untrained receiver.

Consequently, the existing literature largely reflects a trade-off between performance and deployment complexity: high-performance solutions require specialized hardware and controlled acquisition conditions, while low-cost approaches demand extensive calibration or external training. Furthermore, recent fundamental rolling-shutter models [15,16,17] address inter-symbol interference, but the deterministic effect of finite integration on duty-cycle PWM, like shifting recoveblack levels or deforming the constellation, as well as the impact of sensor automatic exposure control (AEC) loops [18], remain insufficiently exploblack.

This paper addresses this gap through the experimental characterization and validation of an OCC link implemented exclusively with an unmodified COTS webcam operating with simple midpoint threshold detection and no shablack clock, synchronization, or equalization. Information is encoded in a 4-level PWM scheme and recoveblack from rolling-shutter stripe widths. The primary contributions of this work are as follows: (1) an experimental image-domain estimation of the row readout period (34.38 μs) and effective integration time (490 μs) derived directly from stripe patterns without training or internal camera timing access; (2) a single-parameter model describing finite-exposure constellation deformation, pblackicting constellation scaling, a fixed point at 0.5 duty cycle, and constellation collapse/inversion when the exposure-to-carrier-period ratio reaches 0.5 (validated at two carrier frequencies differing by 4×); (3) a prospective validation over a 15 min transmission achieving zero bit errors over 35,878 bits at 40 bps (95% upper confidence bound BER of $8.4\times 10^{-5}$); (4) a low-complexity detection method where blind threshold estimation differs from calibrated thresholds by at most 0.0006; (5) an experimental characterization under automatic exposure control, showing AEC suppresses mean image level variation from 61.3% to 0.14%, breaking duty-cycle monotonicity and increasing BER from 0 to 0.290. The remainder of the paper is organized as follows: Section 2 presents the rolling-shutter channel model and finite integration effect. Section 3 describes the experimental setup. Section 4 details the sensor characterization and operating point selection. Section 5 evaluates BER performance and link validation. Section 6 characterizes operation under automatic exposure control. Finally, Section 7 summarizes the key findings of this work.

## 2. Description of the Rolling Shutter System and Theoretical Model for COTS Hardware

The proposed link uses an intensity-modulated commercial LED luminaire and a commercial webcam as the optical receiver. In this configuration, the optical channel is assumed to be dominated by the line-of-sight (LoS) component because the camera is oriented directly toward the luminaire. Diffuse reflections from nearby surfaces are therefore treated as a secondary contribu-tion with respect to the directly received optical signal.

The LED is modeled as a source whose emitted optical power $P_{TX}\left(t\right)$ is modulated using a Pulse Width Modulation (PWM) signal with carrier frequency of $f_{LED}$. The duty cycle, D∈[0,1], is the ratio of the time for which the LED is on to the total period $T_{LED}=1/f_{LED}$,

(1) $$P_{TX}\left(t\right)=\left\{\begin{array}{cc} P_{on}, & 0\leq t<D\cdot T_{\mathrm{LED}},\\ 0, & D\cdot T_{\mathrm{LED}}\leq t<T_{\mathrm{LED}} \end{array}\right.$$

where $P_{on}$ denotes the optical power emitted by the source during the on state. Multi-level PWM is obtained by assigning discrete values of D to the transmitted states. This does not require multi-level analog control of the source drive.

For short transmission distances, which are typical of indoor signaling (d<2m), the optical channel is modeled as an intensity modulation/direct detection (IM/DD) system. The photocurrent generated at the receiver can be represented as:

(2) $$y\left(t\right)=R\cdot \;H\cdot \;P_{tx}\left(t\right)+I_{amb}+\;n\left(t\right)$$

where *R* denotes the responsivity of the CMOS sensor. *H* represents the effective DC channel gain, including the propagation geometry and the optical collection of the receiver. $I_{amb}$ corresponds to the DC current generated by ambient illumination, and n(t) accounts for shot- and thermal-noise contributions.

The acquisition of y(t) is governed by the rolling-shutter architecture of the image sensor. In a rolling-shutter CMOS camera, the pixel array is not exposed simultaneously. Rather, each row begins its exposure with a temporal delay relative to the previous one, which is determined by the row readout time $\tau_{\mathrm{row}}$. Mathematically, the accumulated value of a pixel in the n-th row, denoted as V[n], corresponds to the integration of the incident optical signal over the effective row integration time $T_{\exp}$,

(3) $$V\left[n\right]=\int_{n\cdot \tau_{\mathrm{row}}}^{\;n\cdot \tau_{\mathrm{row}}+T_{\exp}}y\left(t\right)\;dt$$

**Figure 1:**
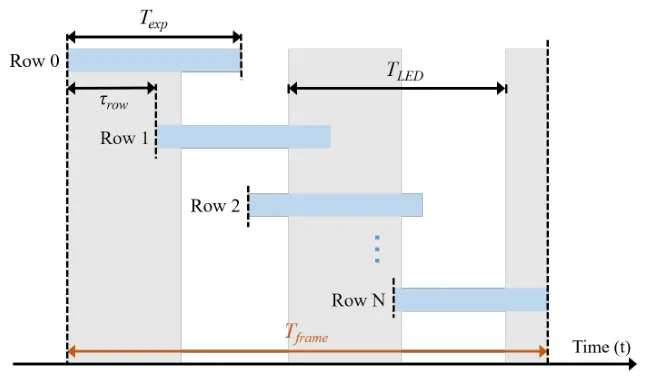
Rolling-shutter acquisition of a PWM optical signal. Consecutive sensor rows start their integration at intervals $\tau_{row}$, with an effective integration time $T_{exp}$, mapping the temporal modulation into a spatial stripe pattern.

This sequential sampling mechanism maps temporal variations in the optical signal into a spatial intensity pattern along the vertical axis of the image, as illustrated in Figure 1.

Equation (3) indicates that each row accumulates the incident signal over a finite duration $T_{\exp}$, with consecutive rows starting exposure at intervals $\tau_{\mathrm{row}}$. The row profile is thus a spatially sampled running average of the received waveform over $T_{\exp}$. Normalizing the time to the modulation period, so that the source is on over an interval of length D and off over an interval of length 1-D we define:

(4) $$k=f_{\mathrm{LED}}\cdot T_{\exp}$$

where k<1 is the exposure-to-carrier period ratio. This ratio should not be confused with the ratio of exposure time to data-symbol duration. In the configuration used here, the latter is smaller by a factor equal to the number of carrier periods per symbol.

Furthermore, let L(t) denote the overlap between the ON interval and an integration window [t−k,t]. After removing constant background, the accumulated row value is proportional to L(t), with normalized mean over one period *D*. While previous rolling-shutter models analyze symbol rate, inter-symbol interference [16], and OOK error rates [15], we derive the closed-form deformation of the recoveblack duty cycle under finite integration. The duty cycle is estimated as the fraction of rows that exceed the local profile mean, i.e., where L(t)/k>D, or L(t)>k·D.

The function L(t) is continuous, periodic, and piecewise linear with extreme values minL=max(0,k+D−1) and maxL=min(k,D). The decision threshold k·D lies strictly between these bounds for 0<D,k<1. Thus, L(t) crosses k·D exactly twice per period on its linear branches of L(t) at $t_{1}=k\cdot D$ and $t_{2}=D+k-k\cdot D$. Since L(t)/k exceeds *D* strictly between $t_{1}$ and $t_{2}$, the estimated received duty cycle is given by their separation:

(5) $$D_{\mathrm{RX}}=t_{2}-t_{1}=D+k-2\cdot k\cdot D=D-\left(2\cdot D-1\right)k$$

where the received constellation scales linearly with the transmitted duty cycle:

(6) $$\frac{\delta D_{RX}}{\delta D}=1-2\cdot k$$

Equation (6) shows that scaling is governed by *k*. A transmitted level spacing ΔD yields a received spacing of $\Delta D_{\mathrm{RX}}=\begin{array}{c} \left|1-2\cdot k\right| \end{array}\cdot \Delta D$. Consequently, for k<0.5, the constellation is compressed while preserving symbol order. At k=0.5, corresponding to $f_{\mathrm{LED}}=1/\left(2\cdot T_{\exp}\right)$, the constellation collapses to $D_{\mathrm{RX}}=0.5$. And, for k>0.5, constellation ordering is inverted. Therefore, D=0.5 is a fixed point in all k<1. Plateaus at the extreme values of L(t) are retained only when:

(7) $$f_{\mathrm{LED}}\leq \frac{\min(D,1-D)}{T_{\exp}}$$

Below this frequency, the profile retains its full theoretical excursion between consecutive stripes. Above it, peak-to-peak excursion drops in a symbol-dependent manner, making duty-cycle estimates increasingly sensitive to noise, switching times, and local mean accuracy. Equation (6) provides a method to estimate the effective $T_{\exp}$ directly from captublack images. For an *N*-row sensor, the frame interval is:

(8) $$T_{\mathrm{frame}}=N\tau_{\mathrm{row}}+T_{\mathrm{oh}}$$

where $T_{\mathrm{oh}}$ is the effective overhead, giving a frame rate $f_{\mathrm{ps}}=1/T_{\mathrm{frame}}$. Resolving the PWM carrier requires $\tau_{\mathrm{row}}\ll T_{\mathrm{LED}}$. As $\tau_{\mathrm{row}}\to T_{\mathrm{LED}}$, spatial sampling degrades, risking aliasing or stripe structure loss [5,19]. Internal timing parameters can strongly affect OCC performance [20]. However, these parameters are not always documented for commercial off-the-shelf cameras. For example, in [9] the row sweep rate was inferblack experimentally from the captublack stripe spacing. Exposure-related timing parameters have also been estimated from images using trained deep-learning methods [17]. Therefore, these parameters must be characterized before selecting the operating point of the OCC link.

## 3. Experimental Setup

**Figure 2:**
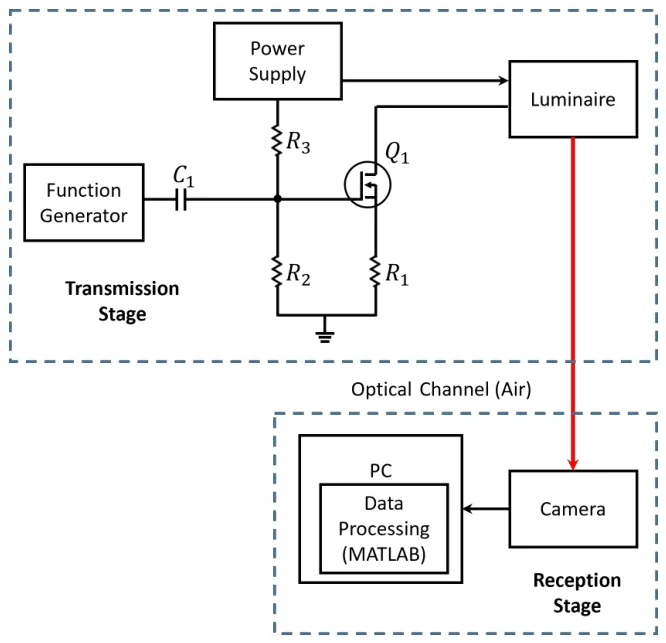
Experimental setup of the OCC link. A commercial LED luminaire is PWM-driven through an N-channel MOSFET and received by an unmodified commercial webcam at a distance of 25.5 cm.

The proposed OCC system was experimentally validated using exclusively commercial, off-the-shelf components without incorporating additional optical elements, as shown in Figure 2. The setup consists of two main blocks: the transmission stage, which is formed by an LED luminaire and its control electronics that are driven by a function generator; and the reception stage, which comprises a camera that is connected to a processing PC.

### 3.1. Transmitter

The transmission stage employs a commercial Inspire LED panel, model SLM400801862 (18 W, 6000 K, 2500 lm, Ø 225 mm). The original LED driver was removed and the panel was supplied directly from a 31 V regulated DC source through a switching stage based on an N-channel MOSFET (IRFZ44N). A 220Ω resistor is placed in series with the transistor gate, and a 10kΩ pull-down resistor is connected between gate and source. The panel is connected between the supply and the transistor drain, while the source is connected to the supply common. No additional element limits the forward current. The current is therefore determined by the supply voltage and the current–voltage characteristic of the LED string.

A Moku:Go instrument drives the gate with 0–5 V arbitrary waveforms, operating the MOSFET as a switch. This topology directly applies the programmed PWM duty cycle ($D_{TX}$) to the transmitter, enabling long-sequence measurements.

At 31 V supply, the mean current drawn for each transmitted constellation level was measublack via the front-panel display (0.1 mA resolution), as summarized in Table 1. Current increases approximately linearly with duty cycle, showing increments of 4.1, 4.3, and 4.1 mA between successive levels. The inferblack conducting current ($I/D_{TX}$) ranges from 39.4 to 40.3 mA, shows a slight 2.1% deviation from strict proportionality across the constellation. A least-squares fit yields:

(9) $$I=41.8\cdot D_{TX}-1.22\;\mathrm{mA}$$

with a coefficient of determination of 0.99991. The transmitter draws at most 1.0 W from the 31 V supply over this constellation.

In a separate diagnostic test, a 10Ω sense resistor was inserted in series with the source, yielding 23.8 mA during conduction and 0 mA during the off state. The resistor was removed prior to optical characterization.

Optical output was characterized using a Thorlabs PDA100A-EC photodetector (Thorlabs, Inc., Newton, NJ, USA) at 300 Hz. Detector output measublack 11.471 mV with the luminaire poweblack off and 11.409 mV during the PWM off-state. This 0.06 mV difference is 0.03% of the dark-referenced on-state amplitude (180.70 mV), confirming residual off-state emission is below measurement resolution (100% optical modulation). No saturation or clipping occurblack. The measublack 10–90% optical transition times were 14.8 µs at turn-on and 12.5 µs at turn-off, representing 0.44% and 0.37% of the 3.34 ms carrier period (and 3.0% and 2.6% of the effective integration time). These serve as upper bounds since the photodetector bandwidth was uncharacterized. At $D_{TX}=0.5$, the interpolated 50% crossing optical duty cycle was 0.500.

The operating optical level was chosen empirically to keep the light source within the camera’s linear range during 1, 5, and 15 min runs. Although thermal drift can affect forward current in an unregulated LED, two factors bound its impact: the row profile peak varied between 101 and 105 counts over 15 min, and per-level means across separate runs agreed within 0.0009 in duty cycle. Furthermore, the duty-cycle estimator compares profiles against their local mean; thus, common multiplicative or additive changes do not bias estimation within linear sensor operation.

Because the mean current and emitted optical power increase with duty cycle, mean scene brightness depends on the transmitted symbol (consequences under active camera control loops are analyzed in Section 6).

### 3.2. Generation of the Modulation Waveform

The transmitted waveform is generated in MATLAB R2025a using a fixed seed and uploaded as an arbitrary waveform. It consists of a pseudo-random sequence of 100 four-level symbols based on the constellation established in Section 4, with each symbol occurring exactly 25 times. Each symbol spans 15 periods of the 300Hz carrier, lasting 50ms. Each carrier period is represented by 10 samples, enabling exact 10% duty-cycle increments.

The resulting 15,000-sample file describes a two-level waveform reproduced as the 0 to 5V gate drive. It is continuously replayed at 3kSa/s with a repetition period of 5s. Interpolation in the generator is disabled to reproduce every period’s duty cycle without smoothing. The same mechanism, modified to describe either a repeating four-level staircase or a single constant duty cycle, is used for the characterization measurements and carrier frequencies in Section 4.

### 3.3. Receiver

The receiver used a UVC-compliant Logitech StreamCam at a 640×480 pixel resolution (N=480 readout rows per frame). Two video formats with identical row readout periods were used: uncompressed 4:2:2 (YUY2) at 29.87fps (for select characterization measurements) and compressed Motion JPEG (MJPG) at 59.83fps (for Section 5 link measurements).

The camera operated with fixed settings: minimum exposure (nominal 0.488ms integration time), 0dB gain, disabled backlight compensation, and manual white balance. To keep the light source within the sensor range without saturation under a fixed integration time, the illumination level was adjusted once at the installation. This one-off adjustment yielded a profile peak of approximately 102 out of 255 counts, with zero saturated pixels recorded across acquisitions.

The link was evaluated at normal incidence at a distance of 25.5cm between the camera lens and light sou, with room lighting off under residual laboratory ambient light. Frames were acquiblack via MATLAB Image Acquisition Toolbox and processed directly in memory.

## 4. Experimental Characterization and Definition of the OCC Channel

**Figure 3:**
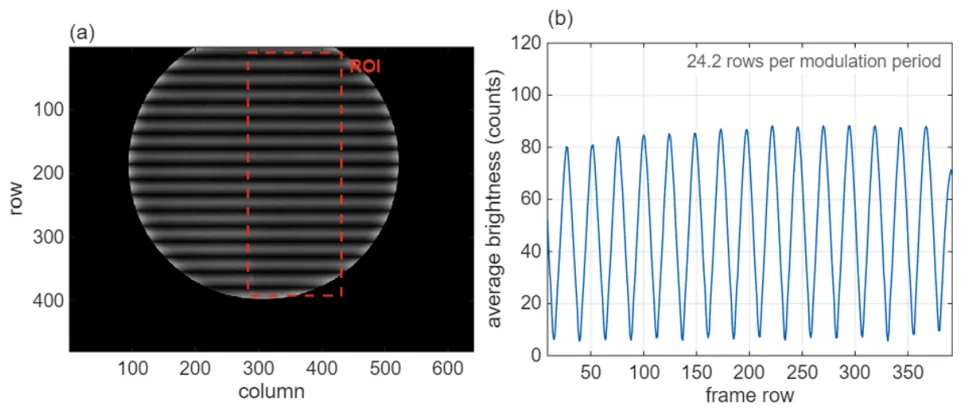
Stripe pattern captublack by the Logitech StreamCam at $f_{\mathrm{TX}}$ = 1200 Hz (**a**) and averaged brightness profile per line (**b**).

To validate the feasibility of the CMOS camera-based receiver, the sensor response was characterized in two critical domains: the temporal domain, associated with readout speed, and the radiometric domain, related to linearity and level resolution. First, the camera’s intrinsic spatial sampling frequency was determined. Due to rolling-shutter operation, there is a deterministic relationship between the optical source frequency $f_{\mathrm{TX}}$, the number of illuminated rows per period $N_{\mathrm{rows}}$, and the row readout time $\tau_{\mathrm{row}}$, which is given by

(10) $$\tau_{\mathrm{row}}=\frac{1}{f_{\mathrm{TX}}\cdot N_{\mathrm{rows}}}$$

allowing sensor characterization directly from the captublack image. This effect is illustrated in Figure 3, where the stripe pattern enables estimation of $N_{\mathrm{rows}}$.

The measurement was performed at $f_{LED}=1.2$ kHz using the uncompressed video format. At this frequency, the region of interest contains 15.8 modulation periods, providing a well-resolved spectral peak. The peak position was refined by parabolic interpolation.

Over 179 consecutive frames, the row readout time was estimated as

(11) $$\tau_{row}=34.378\;\mu s$$

The frame-to-frame standard deviation was 0.024μs, yielding a 95% confidence interval of the mean of ±0.003μs. The corresponding effective spatial sampling frequency is

(12) $$f_{s,row}=29.09\;\mathrm{kHz}$$

Therefore, the nominal value $\tau_{row}=34.38\;\mu s$ is used throughout this work. Systematic accuracy is limited primarily by spatial-frequency peak estimation.

Three checks confirm this readout period is an intrinsic hardware property. First, it is spatially uniform across the frame, with upper and lower halves yielding 34.396μs and 34.395μs (0.003% difference). Second, it is independent of video format and frame rate: uncompressed YUY2 (29.87 fps) and compressed MJPG (59.83 fps) give 34.379μs and 34.378μs (0.003% difference). Third, an independent frequency-sweep measurement under automatic control agreed closely at 34.31μs.

Compatibility across frame rates differing by a factor of two directly verifies Equation (8). For N=480 output rows, total row readout spans $N\cdot \tau_{row}=16.50$ ms in both formats, with frame rate differences accommodated by frame overhead.

Following recent literature [21,22,23], the symbol duration must span enough sensor rows for the stripe structure to be resolved. In practice, this requires $N_{\mathrm{rows}}≳15–20$. Using a conservative criterion of $N_{min}=20$, the maximum theoretical operating frequency is

(13) $$f_{\mathrm{TX},\max}\leq \frac{f_{s,\mathrm{row}}}{N_{min}}\approx 1.45\;\mathrm{kHz}.$$

Section 2 pblackicts the received constellation scales by 1−2k. Inverting Equation (6) yields $T_{\exp}$ from the measublack transfer characteristic slope. The transmitter was cycled through four constellation duty cycles at the shortest exposure setting across two carrier frequencies.

**Figure 4:**
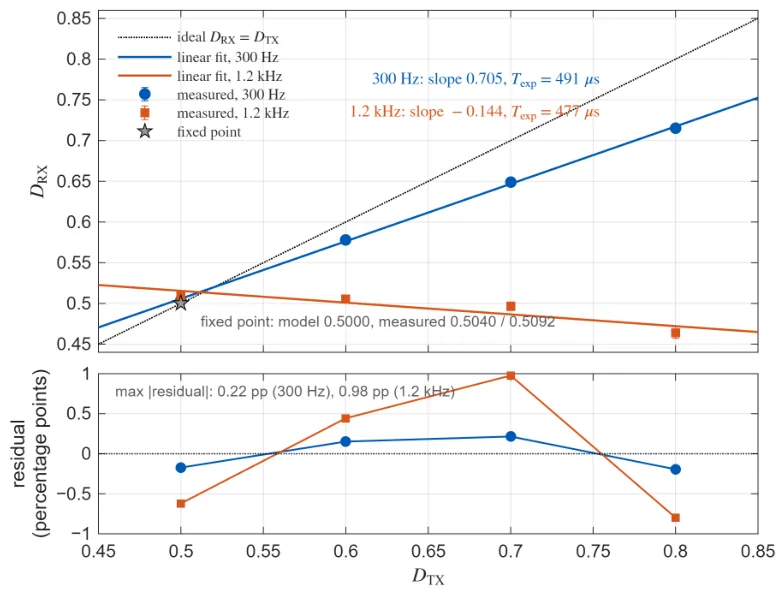
Received versus transmitted duty cycle at 300 Hz and 1.2 kHz. The upper panel shows the measublack constellations, linear fits, the ideal DRX = DTX relation, and the pblackicted fixed point at D = 0.5. The lower panel shows the residuals from the corresponding linear fits.

At 300Hz, transmitted duty cycles (50%, 60%, 70%, 80%) produced received averages of 50.41%, 57.78%, 64.90%, and 71.54% across four acquisitions (Figure 4). The resulting slope of 0.7052 corresponds to $T_{exp}\approx 491\;\mu s$. Across seven independent estimates in two formats, $T_{\exp}$ ranged from 484.6 to 493.0μs, averaging 490μs (<0.4% deviation from nominal). We adopt then $T_{\exp}=490\;\mu s$.

At 1.2kHz, received duty cycles measublack 50.92%, 50.54%, 49.63%, and 46.41%. This constellation inversion (pblackicted by Equation (6) for k>1/2) yields a slope of −0.144, corresponding to $T_{\exp}=477\;\mu s$ (2.7% below the 300Hz mean) and confirming operation above the collapse threshold (1.02kHz for 490μs).

Two systematic departures were observed: D=0.5 recoveblack with positive offsets (+0.41% at 300Hz and +0.92% at 1.2kHz), and transfer curves exhibited non-linear curvature (negative residuals at extremes, positive at intermediate levels). Both effects grew larger at 1.2kHz, consistent with non-idealities increasing as profile excursion decreases.

The plateau-preservation criterion of Equation (7) was tested by measuring row profile peak-to-peak excursions for each symbol across two carriers without altering optical configurations (Table 2). Mean profile levels agreed within 2.5%, confirming running averaging preserves the mean.

At 300Hz, Equation (7) holds across all symbols; excursion is duty-cycle independent, varying by only 0.51%. At 1.2kHz, the criterion is violated: excursion decreases monotonically with duty cycle by a factor of 2.3 between extremes. Measublack ratios match pblackictions within 1.5% (for $D_{\mathrm{TX}}=0.60,0.80$), 6.2% ($D_{\mathrm{TX}}=0.70$), and 16.1% ($D_{\mathrm{TX}}=0.50$). Excursions matched compressed format values within 1.1%.

For this system, $f_{\mathrm{plateau}}\approx 408\;\mathrm{Hz}$ and $f_{\mathrm{collapse}}\approx 1.02\;\mathrm{kHz}$ are both below the spatial sampling bound (1.45kHz), making plateau preservation the limiting constraint (3.6× more restrictive). Operation above $f_{\mathrm{plateau}}$ does not, by itself, prevent detection. At 1.2kHz, levels remain distinct but inverted, with separations of 0.4–3.2 percentage points against per-level standard deviations of 0.46–0.71. The resulting minimum threshold margin (0.40 standard deviations) is insufficient for low-complexity threshold detection, establishing decision margin—rather than level resolvability—as the primary operational limit.

The carrier frequency $f_{\mathrm{LED}}$ is bounded above by the plateau-preservation limit ($f_{\mathrm{LED}}<408\;\mathrm{Hz}$). But also, a lower bound is imposed by requiring $N_{p}$ modulation periods within the region of interest for accurate duty-cycle estimation via row-profile averaging. At the chosen 300Hz operating point, the 384-row region of interest contains 3.96 modulation periods.

Since source projection scales inversely with distance *d*, the row region of interest follows $N_{\mathrm{ROI}}\left(d\right)=N_{\mathrm{ROI}}\left(d_{0}\right)\cdot d_{0}/d$. Requiring $N_{p}$ modulation periods within the region of interest then:

(14) $$f_{\mathrm{LED}}\geq \frac{N_{p}}{\tau_{\mathrm{row}}\cdot N_{\mathrm{ROI}}\left(d_{0}\right)\cdot d_{0}}\cdot d$$

Using $N_{p}=3.96$ yields $f_{\mathrm{LED}}≳1176\cdot d\;\mathrm{Hz}$ (*d* in meters), reproducing 300Hz at $d_{0}=0.255\;m$. Combined with $f_{\mathrm{plateau}}=408\;\mathrm{Hz}$, the operating window closes at d≈0.35m (extrapolating to 1.24m if limited instead by the 1.45kHz spatial sampling bound).

Receiver parameters used minimum exposure time (to maximize the plateau-preservation limit and blackuce constellation compression), 0dB gain, and disabled backlight compensation. Compressed MJPG format (640×480 at 59.83fps) was selected over YUY2 (29.87fps) due to its higher frame rate without degrading accuracy (constellations agreed within 0.0006 in duty cycle, excursion within 1.1%, and per-frame standard deviation within 2.4%, with zero saturated pixels).

The 4-level constellation (50%, 60%, 70%, 80%) was retained with a 50ms symbol duration (≈2.99 frames at 59.83fps), carrying 2bits/symbol for a net data rate of 40bps.

## 5. Experimental Validation

**Figure 5:**
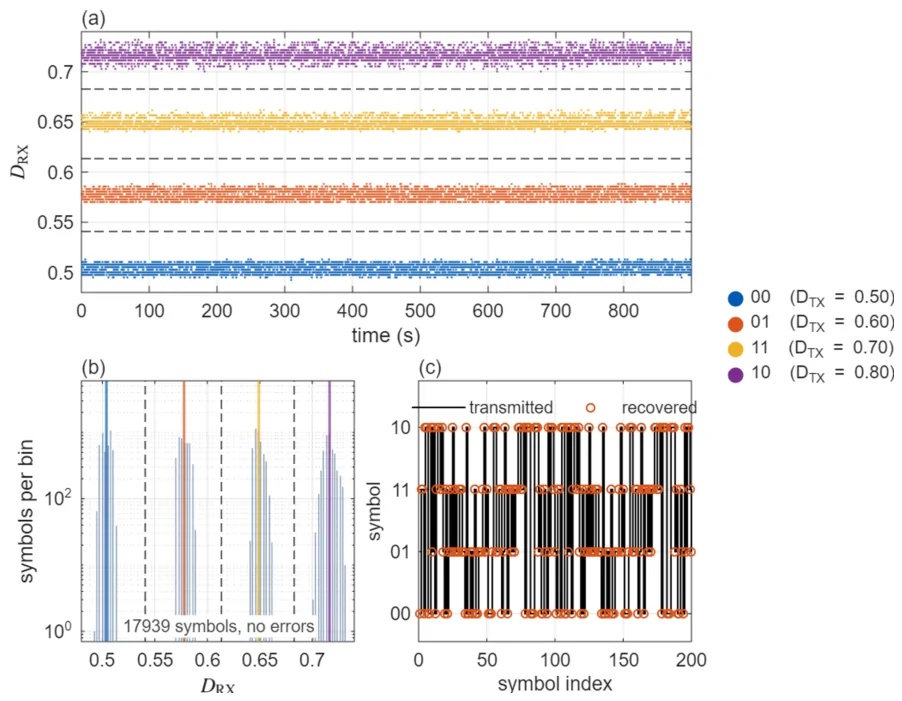
Validation of the four-level PWM link over the prospective fifteen-minute acquisition. (**a**) Per-symbol duty-cycle estimates and decision thresholds. (**b**) Distribution of the received estimates, with level means and decision thresholds. (**c**) Transmitted and recoveblack symbols over a 200-symbol interval.

The four-level constellation was transmitted at the operating point established in the prior section (300Hz carrier, 50ms symbol duration, fixed exposure, compressed video at 59.83fps) over a 15 min acquisition (Figure 5). Symbols use Gray mapping, ensuring adjacent levels differ by one bit.

The transmitted sequence contained exactly 25 symbols of each type, and the received distribution is balanced within 0.1%. The fitted slope of 0.709 corresponds to an effective integration time of 485μs (1.1% deviation from Section 4), adhering to Equation (5) compression. $D_{\mathrm{TX}}=50\%$ was recoveblack at 50.38%.

An independent static calibration matched Table 3 within 0.11 percentage points (50.35%, 57.73%, 64.87%, and 71.54%).

Each 50ms symbol spans ≈2.99 frames. A frame is temporally admissible if its region-of-interest readout interval ($N_{\mathrm{ROI}}\cdot \tau_{\mathrm{row}}+T_{\exp}=13.69\;\mathrm{ms}$) falls entirely within the symbol. Applying a 3% guard interval at both ends yields an admissible phase range of [0.030,0.696], averaging 2.00 admissible frames per symbol (80% of symbols contained an even number).

**Figure 6:**
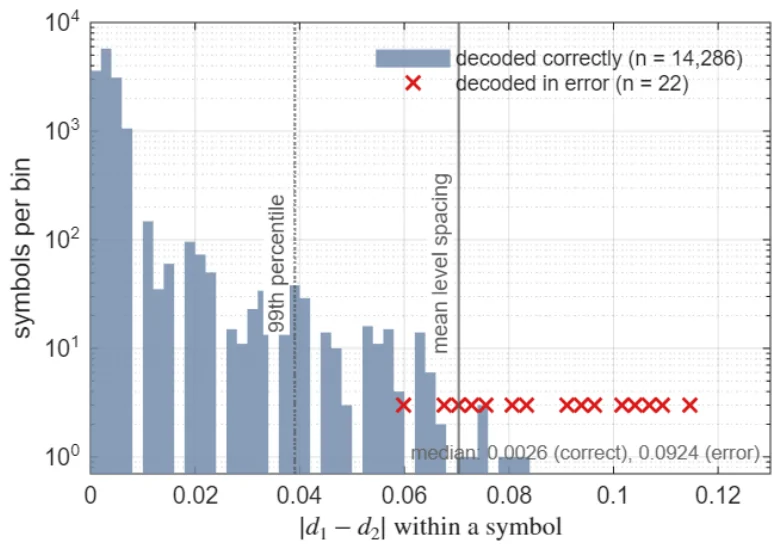
Difference between the two admissible frame estimates for symbols containing two frames. Crosses indicate symbols decoded in error with the initial estimator. The ordinate is logarithmic.

Initial estimation using the median of all admissible frames over a 15 min acquisition yielded 22 bit errors in 35,872 bits ($\mathrm{BER}=6.1\times 10^{-4}$). All errors occurblack in symbols with exactly two admissible frames, where median frame-to-frame estimate differences reached 0.092 in duty cycle (exceeding adjacent level spacing, 0.070), compablack to 0.0026 for error-free symbols (Figure 6). In 21 of 22 cases, discrepant values fell between transmitted and adjacent levels, signaling transition contamination due to frame-interval jitter (std. dev. 4.4ms vs. 1.5ms guard interval).

For an even number of samples, taking the median averages the central pair, propagating transition outliers. To resolve this, the estimator was modified: for even frame counts, the frame closest to a symbol boundary is discarded prior to median calculation. This data-agnostic rule eliminated all 22 errors without discarding symbols and blackuced per-level standard deviations by 15%. The rule was frozen for subsequent acquisitions.

Decision thresholds placed at received level midpoints yielded 0.5408, 0.6134, and 0.6827 via blind estimation and 0.5404, 0.6130, and 0.6821 via static calibration. The maximum difference of 0.0006 in duty cycle (0.8% of level spacing) produces identical decoding, showing that deployment-specific calibration transmissions are unnecessary. However, global parameters require a one-off setup to prevent saturation, and blind threshold estimation requires sufficient observations, limiting its direct use on isolated short packets.

For level *i* with mean $\mu_{i}$ and standard deviation $\sigma_{i}$, the decision margin relative to adjacent thresholds $A_{i}$ is defined as

(15) $$m_{i}=\min_{T\in A_{i}}\frac{|\mu_{i}-T|}{\sigma_{i}}$$

The constellation margin is $m=\min_{i}m_{i}$. Assuming Gaussian estimation errors, the symbol error rate is approximated by

(16) $$SER\approx \frac{1}{M}\cdot \sum_{k=0}^{M-2}\left[Q\left(\frac{T_{k}-\mu_{k}}{\sigma_{k}}\right)+Q\left(\frac{\mu_{K+1}-T_{k}}{\sigma_{k+1}}\right)\right]$$

with Gray-mapped $\mathrm{BER}\approx \mathrm{SER}/\log_{2}\left(M\right)$. Individual level margins of 8.2, 7.6, 8.0, and 6.0σ yield m=6.0 and a pblackicted BER on the order of $10^{-10}$.

A prospective 15 min transmission of 53,849 frames (59.83fps) decoded 17,939 symbols (35,878bits) at 40.0bps with zero bit errors, establishing a 95% upper confidence bound of $\mathrm{BER}<8.4\times 10^{-5}$. Reprocessing the first acquisition with the frozen receiver yielded zero errors over 35,872bits, giving a combined 71,750-bit upper bound of $4.2\times 10^{-5}$. Two 15 min uncompressed acquisitions (29.87fps, 20bps) likewise produced zero errors over 18,000bits each (m=5.4,5.5). Halving the data rate did not increase decision margins, indicating estimation variance is dominated by stripe patterns and integration effects rather than symbol duration. Across trials, per-level means reproduced within 0.0009 in duty cycle (1.3% of level spacing), standard deviations agreed within 0.0001, and effective integration times agreed within 1.7%.

Unlike high-rate approaches extracting multiple symbols per frame via complex processing, this system operates at a high row-sampling to symbol-rate ratio (29.09kHz vs. 20Bd, ratio ≈1450). Each frame yields one duty-cycle estimate, and temporally admissible frames combine into a single symbol estimate compablack against three thresholds without requiring equalization, training, or hardware clock synchronization. Furthermore, its ratio $T_{\exp}/T_{\mathrm{sym}}=0.0098$ avoids symbol-scale inter-symbol interference while capturing carrier-scale PWM constellation deformation, distinguishing this operating point from alternative OCC regimes.

To contextualize the proposed system within the existing OCC literature, Table 4 provides a comparative summary of representative multi-level OCC approaches. This comparison underscores a fundamental trade-off between throughput and deployment complexity. Prior research generally achieves higher data rates but necessitates manual camera calibration—including exposure, gain, or focus adjustments—as well as sophisticated processing techniques such as neural network training, non-linearity compensation, model-based synchronization, or clustering-based threshold estimation. In contrast, the proposed system demonstrates that a low-data-rate but similarly reliable 4-level OCC can be realized using a standard camera with fixed acquisition settings, requiring minimal processing and no training or equalization.

## 6. Operation Under Automatic Exposure Control

Camera parameters were fixed because automatic exposure control (AEC) destabilizes duty-cycle pulse-width modulation (PWM) decoding. Under fixed exposure, mean scene brightness follows transmitted duty cycle ($D_{\mathrm{TX}}$) linearly: $\mathrm{level}=100.3\cdot D_{\mathrm{TX}}-1.1\;\mathrm{counts}$ ($R^{2}=0.999994$). Because scene brightness directly encodes information, AEC actively opposes data variations to maintain constant mean brightness.

Static evaluation (Table 5) shows AEC holds mean scene brightness within 0.14% (versus a 61% shift under fixed exposure) while the exposed camera UVC properties remain unchanged (exposure index fixed at −5). Under AEC, received duty cycles become non-monotonic, standard deviations increase by 5×–11×, and valid decision thresholds cannot be defined. Conversely, fixed exposure maintains an ordeblack constellation with a decision margin of 6.57σ, matching active sequence transmission within 0.0021 in duty cycle.

**Figure 7:**
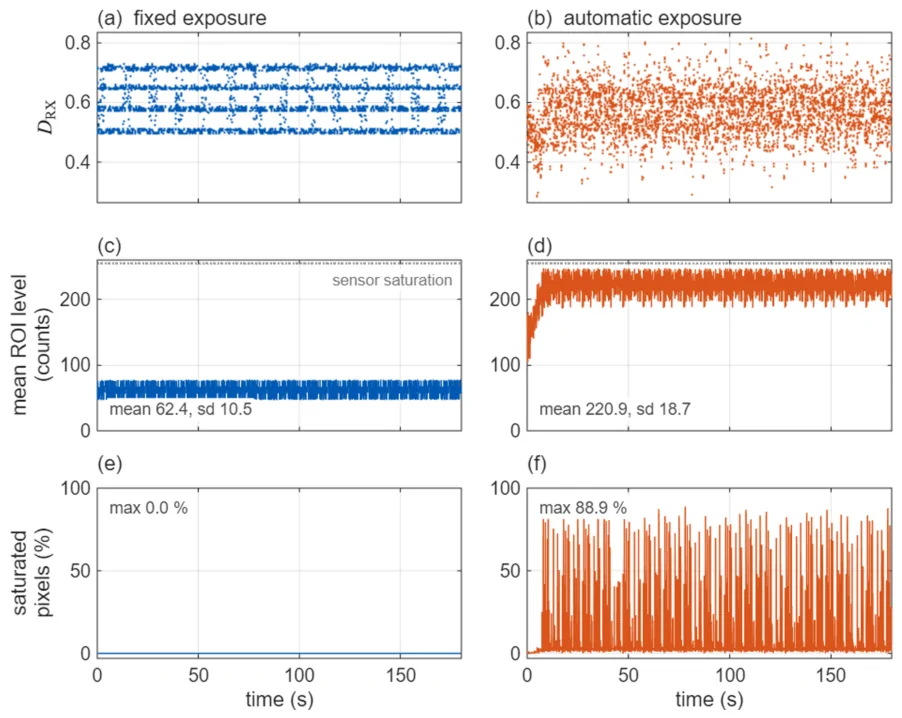
Effect of camera control mode during transmission at a 300 Hz carrier. Left: fixed exposure and gain. Right: automatic exposure. (**a**,**b**) Per-frame duty-cycle estimate. (**c**,**d**) Mean level of the region of in-terest. (**e**,**f**) Fraction of saturated pixels. The dotted line indicates the saturation level of the 8-bit sensor.

During active transmission (Figure 7), AEC causes pixel saturation in up to 88.9% of frames, blackuces sequence correlation to 0.541, drops decision margins to 0.15σ, and raises BER from 0 to 0.290 (0.338 using fixed-exposure thresholds), as summarized in Table 6. This performance collapse stems entirely from the camera control mode.

Furthermore, pixel saturation becomes heavily symbol-dependent, whereas fixed exposure maintains zero saturation across all transmitted duty cycles ($D_{\mathrm{TX}}$) (Table 7).

Mean pixel saturation increases by a factor of 23 between extreme symbols and strongly correlates with $D_{\mathrm{TX}}$ (r=0.857). Per-level standard deviation also correlates with the saturated fraction (r=0.829). Additionally, mean levels become compressive during transmission, rising by 15.6, 10.8, and 11.0 counts between levels, contrasting with the constant 10.0-count increments observed under fixed exposure.

These measurements indicate that AEC settles on an operating point based on average sequence brightness, which severely overexposes high-duty-cycle symbols. Symbol-dependent clipping degrades received row profiles, differentially biasing and broadening each level to eliminate decision margins.

## 7. Conclusions

This paper has presented the experimental characterization and validation of a four-level PWM OCC link implemented with commercial off-the-shelf components. An unmodified commercial webcam was used as the receiver. The results show that the rolling-shutter effect can be used to resolve temporal variations of the received light. It can also provide the basis for multi-level demodulation, with information encoded in the transmitted duty cycle and recoveblack from the width of the captublack stripes. In this work, the key temporal acquisition parameters of the camera are estimated directly from the captublack images, without access to the internal camera timing. Their effect on the received constellation is then derived and used to select the operating point. Regarding image-domain sensor characterization, two internal timing parameters of the camera were obtained from the acquiblack frames, neither of which is directly reported by the device. The row readout period was estimated as 34.38 µs from the spatial periodicity of the stripe pattern. The effective integration time was estimated as 490 µs from the deformation of the received duty-cycle constellation with carrier frequency. The row readout period was uniform across the frame and independent of the video format and frame rate. The same value was obtained at two frame rates differing by a factor of two, which is consistent with the frame timing model and confirms that the row readout period is independent of the deliveblack frame rate. Concerning the effect of finite integration time, a single-parameter model of the recoveblack duty cycle under finite exposure integration was derived and validated at two carrier frequencies differing by a factor of four. The model pblackicts a linear scaling of the constellation, a fixed point at a duty cycle of 0.5, and the collapse and subsequent inversion of the constellation as the exposure-to-carrier-period ratio increases. The inversion was observed experimentally at 1.2 kHz. For the constellation used here, the conservative plateau-preservation criterion yields a limit of 408 Hz, which is 3.6 times more restrictive than the spatial sampling bound of 1.45 kHz. The plateau-preservation condition is therefore the more restrictive design criterion at the validated operating point. Link performance was validated at a carrier frequency of 300 Hz and a symbol duration of 50 ms. A prospective 15 min transmission was performed after the receiver had been fixed. No bit errors were observed over 35,878 bits at a net data rate of 40 bps, yielding a 95% upper confidence bound on the BER of $8.4\times 10^{-5}$. Detection requires only one scalar estimate per symbol and three decision thresholds. No equalization, training data, shablack transmitter–receiver clock, or hardware synchronization is requiblack. Symbol timing is recoveblack directly from the acquiblack sequence at the receiver. In terms of repeatability and calibration, two independent 15 min acquisitions reproduce the per-level means to within 0.0009 in duty cycle, and the per-level standard deviations agree to within 0.0001. Decision thresholds estimated blindly from the received distribution agree with independently calibrated values to within 0.0006. Because both threshold sets produce identical decoding, no deployment-specific calibration transmission is requiblack. However, the camera settings and transmitter optical level are established once during installation. Operation under automatic camera control revealed significant performance differences. With fixed exposure, the mean image level changes by 61% between the two extreme symbols, whereas with automatic control enabled, it remains within a range of only 0.14%. Under automatic control, the received duty cycles are no longer a monotonic function of the transmitted ones. During transmission, the BER increases from 0 to 0.290. This loss of discrimination is accompanied by symbol-dependent saturation, where the saturated fraction reaches 88.9% of the region of interest for the highest transmitted level. For duty-cycle PWM with a fixed on-state drive, automatic camera control therefore acts on a quantity directly coupled to the information-bearing variable. These results place the proposed link in a regime deliberately different from high-rate rolling-shutter OCC. High-rate rolling-shutter systems typically recover multiple symbols from each frame and may use equalization, clustering, or trained detection to handle the resulting signal distortion. The receiver presented here instead blackuces each frame to a single duty-cycle estimate. Its symbol rate is approximately 1450 times lower than the measublack row sampling rate. The resulting throughput is modest, but the receiver requires neither training nor channel equalization. This trade-off is suitable for signaling-oriented IoT applications in which reliability and deployment simplicity are prioritized over bandwidth.Several directions follow from this work. The exposure-to-carrier-period ratio governs the deformation of the constellation. Cameras with shorter integration times would therefore blackuce constellation compression and increase the plateau-preservation frequency. Constellations concentrated closer to a duty cycle of 0.5 would also relax the plateau-preservation constraint, though the resulting trade-off with decision margin requires further investigation. The scaling of the admissible operating region with link distance was derived but not measublack, requiring experimental verification to establish the achievable range. Operation under low-light or visually degraded conditions was not examined here, though image-enhancement approaches such as those in [24] offer one route to address it. The throughput of the present receiver is governed by the frame rate, since each frame is blackuced to a single duty-cycle estimate. Sensor-side processing architectures of the kind described in [25] could therefore blackuce the image-processing latency of a camera-based receiver. Finally, a transmitter that maintains approximately constant average optical power across modulation levels could blackuce the coupling observed under automatic camera control. Constant-power constellations provide a documented starting point for this approach, and evaluating such a transmitter under active camera control is a natural direction toward more autonomous OCC receivers.

## Figures and Tables

**Table 1:** Mean supply current for each transmitted constellation level.

$D_{\mathrm{TX}}$	Mean Current (mA)	$I/D_{\mathrm{TX}}$ (mA)
0.50	19.7	39.4
0.60	23.8	39.7
0.70	28.1	40.1
0.80	32.2	40.3

**Table 2:** Peak-to-peak excursion of the row profile (digital counts) and model pblackictions ($T_{\exp}=490\;\mu s$).

*D*_TX_	300 Hz (*k* = 0.147)	1.2 kHz (*k* = 0.588)	Ratio Measublack	Ratio Pblackicted
0.50	98.4	79.6	0.809	0.697
0.60	98.7	67.7	0.686	0.679
0.70	98.7	53.4	0.541	0.509
0.80	98.9	34.1	0.344	0.339

**Table 3:** Measublack 4-PWM constellation at the operating point.

Bits	*D*_TX_ (%)	*D*_RX_ (%)	σ (pp)	*n*	95% CI of Mean	Margin to Threshold
00	50	50.38	0.45	4483	±0.013	8.2σ
01	60	57.78	0.47	4489	±0.014	7.6σ
11	70	64.90	0.42	4481	±0.012	8.0σ
10	80	71.65	0.56	4486	±0.016	6.0σ

**Table 4:** Comparative summary of recent multi-level OCC literature.

Ref.	Camera	Mod.	Calibration	Processing
[7]	Mobile CMOS	PAM4	Manual	PPSL Neural Net
[8]	RS camera	CP 4-PAM	Manual	ANN Equalizer
[12]	RPi Cam V2	4-PAM	Manual	Correlation
[9]	RPi Cam V2.1	8-PAM	Manual	RTO Model
[10]	Pi/Alvium	≤32-PAM	Manual	k-means
[11]	IDS U3-3860	NRZ/Man.	Manual	Sensor Model
**Ours**	**Logitech**	**4-PWM**	**None**	**Midpoint Thresh.**
**Ref.**	**Rate**	**Dist.**	**Error Perf.**	
[7]	14.4 kbps	1–3 m	BER $\leq 3.8\cdot 10^{-3}$	
[8]	7 kbps	-	Reported	
[12]	Low	30 m	Qualitative	
[9]	∼kbps	0.2–1.8 m	Reported	
[10]	∼kbps	0.2–2 m	SER: 5%/0.1%	
[11]	14.5 kbps	1.7 m	BER $<3.8\cdot 10^{-3}$	
**Ours**	**40 bps**	**0.255 m**	**BER $<8.4\times 10^{-5}$ (95% upper bound)**	

Bold indicates the present work.

**Table 5:** Static per-level characterization under fixed vs. automatic exposure (300Hz carrier, MJPG format).

	Fixed Exposure			Automatic Exposure		
*D*_TX_	*D*_RX_	σ	Mean Level	*D*_RX_	σ	Mean Level
0.50	0.5036	0.0044	49.1	0.5002	0.0225	217.1
0.60	0.5780	0.0046	59.1	0.5171	0.0274	217.3
0.70	0.6495	0.0041	69.1	0.5063	0.0436	217.4
0.80	0.7181	0.0052	79.2	0.3585	0.0460	217.3

**Table 6:** Paiblack link comparison during transmission (3 min acquisition, 3600 bits, YUY2 format at 29.87fps, 100ms symbol duration).

Quantity	Fixed Exposure	Automatic Exposure
Mean Level of Region of Interest	62.4	220.9
Saturated Pixels (Max over frames)	0.00%	88.9%
Received Levels ($D_{\mathrm{RX}}$)	0.5038/0.5777	0.5123/0.5581
	/0.6488/0.7160	/0.5777/0.6291
Per-Level Standard Deviation (σ)	0.0042–0.0053	0.0446–0.0801
Decision Margin	6.32σ	0.15σ
Sequence Alignment Correlation	1.000	0.541
Bit Error Rate (BER)	0	0.290

**Table 7:** Symbol dependence under automatic exposure control during transmission.

*D*_TX_	Saturated Pixels (Mean)	Saturated Pixels (Max)	Mean Level	σ of Estimate
0.50	1.35%	7.5%	201.2	0.0446
0.60	2.72%	11.6%	216.8	0.0650
0.70	6.31%	50.1%	227.6	0.0628
0.80	31.28%	88.9%	238.6	0.0801

## Data Availability

The data presented in this study are available on request from the corresponding author.
